# The Evolution of the Trained Nurse1Address to the Nurses graduating from the German Hospital, Buffalo, N. Y., 1905.

**Published:** 1905-12

**Authors:** Herman E. Hayd

**Affiliations:** Buffalo, N. Y.


### SELECTIONS.

### The Evolution of the Trained Nurse.[1]

By HERMAN E. HAYD, M. D., M.R.C.S., ENG., Buffalo, N. Y.

[From *Trained Nurse*, November, 1905.]

YOU are now separating yourselves from this institution and severing ties and associations which will always be among the pleasantest memories of your lives, and you are giving up daily companionship with friends and teachers who have contributed much to your education and to the vast storehouse of practical knowledge which will enable you to fill useful lives in the community. Your work is but begun and your real duties and responsibilities have just commenced. Here you have had your superintendent and medical staff to encourage you and to protect you, but soon you will be dependent upon your own exertions and will be compelled to utilize your studies and experiences in the competitive and exacting walks of life. The motives which prompted you to take up the noble profession of nursing must have been generous ones, and you have realized by this time that your life work, if it is to be successful, must be one largely of self-sacrifice and self-abnegation. Few people know how difficult and exacting your duties are, and fewer still

1. Address to the Nurses graduating from the German Hospital, Buffalo, N. Y., 1905.

will grant you that appreciation and reward which you deserve. The nervous tension associated with your work, the long vigil and loss of sleep consequent upon its performance, the tax upon your physical strength and endurance in waiting upon the acutely sick and helpless, too soon make their terrible impressions upon your constitution and render you unfit for the further discharge of your duties. Your professional life, therefore, at best is not a long one, and it behooves you to take early warning that in order to serve others you must also nurse yourselves. Adopt at once habits of regularity and insist upon getting rest, sleep and a certain amount of recreation, amusement and exercise. No matter how important your case may be, let it be early understood that a certain portion of the day must be given to you for outdoor life.

The compensation paid a nurse to-day is ample and is sufficient to meet all her wants and reasonable demands. You will often be called upon to give your services for less than the usually allowed amount, and when the people are poor and deserving respond at once and willingly. The possibilities of your profession are unbounded and the calls which may be made upon your talents and services know no limitations. Your work opens for you all the treasures of an exalted womanhood. Your services will not be confined simply to the sickroom in times of peace and plenty, but they may be demanded during seasons of scourge and famine. You may be called to the battlefield and suffer all kinds of privations—to nurse in fevered hospitals, in tents and in wagons, the soldiers and sailors who have been called in defense of your country and its flag; and perhaps be forced even into the firing line amidst the most sickening sights of carnage and death, to move about as angels of mercy, giving here and there help and comfort to the wounded and dying.

Nature has endowed women with her richest and most honorable instincts and attributes. She has filled your natures with emotions—tenderness, gentleness, sympathy and kindness—and your education as nurses gives you the opportunities to use these noble qualities advantageously. When the Lord created man he exercised a divine fiat: "In the sweat of thy face shalt thou eat bread," and you were to be his companion and helpmate, wife, mother and nurse. Through years of varying social conditions and the unnatural and unfair accumulation and distribution of wealth, and through sickness, misfortune and adversity of the male supporting members of society, necessity has compelled woman to seek employment for a livelihood and to be dependent upon herself for her maintenance and support. Various occupations from time to time have opened their portals to you, and as a result women have become in a large measure the world's breadwinners. As in other fields of labor, so here, there has been a gradual evolution in the development of the so-called nurse. At first her duties and responsibilities were few and small and she was simply an ordinary domestic servant. The necessities and claims of the sick increased her possible usefulness, and then demanded of her more exacting duties and responsibilities, and as a result there was developed a position for a refined and educated woman, who in time sought a technical training, and consequently there was initiated in connection with hospitals various training schools from which men and women were graduated and were given diplomas that guaranteed the standing, excellence and qualifications of the persons who possessed them.

This is the position you find yourself in to-night.

Let us turn for a few moments to the more practical part of our subject and ask ourselves what constitutes a good nurse. Above all, she must be a woman of character and be pure in thought and life. She must possess and must always cultivate a kindly disposition; be patient and be full of forbearance and sympathy. She must have the power to control her temper under the most distressing and often aggravating circumstances; she must be cheerful, bright and entertaining, but at the same time serious and resolute when necessary. She must be tactful, not arrogant and boastful; she must not be too communicative, neither too secretive, but always honest and truthful. The secrets of your patient and the trials of the bedchamber shall be inviolate in your keeping. She must be intelligent and studious; she must be cleanly about herself, and endeavor at all times to be neat and attractive but not fussy. She must be capable, not only in her ministrations upon the sick, but in preparing and serving their food. Present their meals in as dainty and attractive manner as possible; a nice tray with a clean napkin. and dishes garnished with some greens or fresh blossoms. Even in the homes of the poor little delicacies can occasionally be served, and in an appetizing manner; and no matter how poor and humble your patient may be, these little gentle attentions come like sweet messengers of love and hope and bring cheer and tranquility to mind and body.

A nurse with a seriously sick patient should be calm, cheerful and hopeful. These qualities encourage and strengthen and fortify in the desperate fight they are making. She should move about the room quietly, but not stealthily; she should speak plainly and directly, and her manipulations with the patient should be firm and with every evidence of confidence in herself. She should be ready at all times to answer the patients demands and make her feel that the service is given kindly and with tenderness and affection. Don't answer back quickly and in a spirit of retaliation. Don't slam doors and make unnecessary noises either in walking about or doing your several duties. Often your patients will disturb you from your sleep, and unnecessarily, but remember this is their privilege, and you must at once respond to their calls or if possible tactfully soothe them by a kind word. They will tax your patience beyond endurance like a peevish, irritable child, and no matter what you do for them they will demand something else or wish it done in a different way. A quick word or a sharp answer will wound so deeply that your future devotion. no matter how kind and slavish it may be, will not rehabilitate yourself in their confidence and affection. Better resign from the case and have some new blood with fresh patience and new cheer and new hopes in the sickroom, because temperature, increased pulse, loss of appetite and even serious symptoms can be kept up because of these often slight and trivial disturbances. Of course, to serve such a patient will tax all human endurance and understanding, and yet if you always have in your mind an ideal nurse and a noble woman you will establish for yourself such a standard of excellence that there will be fewer complaints and your popularity will increase and your patients will get well and will have no other in attendance in their families, and the doctor will bless and thank you a hundred times for your courage and for your tact, and above all for your successful management of his cases. Be loyal to your friends and to your profession. Protect your fellow nurses. Don't gossip about them or other people or engage yourself in unseemly conversation. no matter what encouragement you may get from your patient, because just as soon as you place yourself on a par with their standard of virtue and morality you detract from your own strength and usefulness.

Uphold your doctor, or resign from the case if you feel that his practice is below your standard of excellence and you cannot serve with him. Do not hint by suggestion or innuendo that Dr. So-and-so does so and so, because there are many different ways in the methods and management of diseases and you are not familiar with all of them, nor have you the knowledge necessary to qualify you to question them. However, it may occur to you in the nursing of a case that your previous experience enables you to teach the medical attendant many things which your technical training has specially fitted you for, such as the administration of baths, packs, douches, etc. He would indeed be a fool who would not accept your suggestions and perhaps wisdom. But be careful to make these remarks in an anteroom, so that the patient's tranquillity of mind and confidence are not disturbed.

Your position in the sickroom, although a noble one, is often an exceedingly difficult and trying and unjust one to fill. The doctor receives the honors and the glory and often they come through your hands. He comes into the room for a few moments and brings sunshine, and when he leaves he often takes with him the only ray that you had kindled and started aglow. Your colossal efforts to sustain and encourage when hope is gone through the wearying weeks and months of slow but sure dissolution are often beautiful, and have more than once made me feel that woman's stream of perpetual beauty and sweetness seems to be inexhaustable. Your reward is small and not commensurate with your deserts, and the only compensation you often have is the consciousness of duty well performed. But your privileges are great. Into your arms the new-born babe is given, and from them it is placed upon the mother's breast; and from your gentle hands comfort is dispensed and light and cheer throughout succeeding periods, and finally when old age comes with its increasing infirmities you can give support and surcease during that long journey—perhaps of pain and suffering—twixt this life and a blessed eternity, and close forever those tired eyelids in the last long sleep of death.

For these noble duties and labors you have to-day consecrated yourselves, and after years in its service it is the hope of your teachers and your training school and hospital that it may be said of you: "Well done, thou good and faithful servant; thou hast been faithful over few things, I will make thee ruler over many things; enter thou into the joy of thy Lord."

493 Delaware Avenue.

